# Sex Differences in the Regulation of Offensive Aggression and Dominance by Arginine-Vasopressin

**DOI:** 10.3389/fendo.2017.00308

**Published:** 2017-11-14

**Authors:** Joseph I. Terranova, Craig F. Ferris, H. Elliott Albers

**Affiliations:** ^1^Center for Behavioral Neuroscience, Neuroscience Institute, Georgia State University, Atlanta, GA, United States; ^2^Department of Psychology, Center for Translational NeuroImaging, Northeastern University, Boston, MA, United States

**Keywords:** gender differences, agonistic behavior, social status, social communication, oxytocin, serotonin, V1a receptors, V1b receptors

## Abstract

Arginine-vasopressin (AVP) plays a critical role in the regulation of offensive aggression and social status in mammals. AVP is found in an extensive neural network in the brain. Here, we discuss the role of AVP in the regulation of aggression in the limbic system with an emphasis on the critical role of hypothalamic AVP in the control of aggression. In males, activation of AVP V1a receptors (V1aRs) in the hypothalamus stimulates offensive aggression, while in females activation of V1aRs inhibits aggression. Serotonin (5-HT) also acts within the hypothalamus to modulate the effects of AVP on aggression in a sex-dependent manner. Activation of 5-HT1a receptors (5-HT1aRs) inhibits aggression in males and stimulates aggression in females. There are also striking sex differences in the mechanisms underlying the acquisition of dominance. In males, the acquisition of dominance is associated with the activation of AVP-containing neurons in the hypothalamus. By contrast, in females, the acquisition of dominance is associated with the activation of 5-HT-containing neurons in the dorsal raphe. AVP and 5-HT also play critical roles in the regulation of a form of social communication that is important for the maintenance of dominance relationships. In both male and female hamsters, AVP acts *via* V1aRs in the hypothalamus, as well as in other limbic structures, to communicate social status through the stimulation of a form of scent marking called flank marking. 5-HT acts on 5-HT1aRs as well as other 5-HT receptors within the hypothalamus to inhibit flank marking induced by AVP in both males and females. Interestingly, while AVP and 5-HT influence the expression of aggression in opposite ways in males and females, there are no sex differences in the effects of AVP and 5-HT on the expression of social communication. Given the profound sex differences in the incidence of many psychiatric disorders and the increasing evidence for a relationship between aggressiveness/dominance and the susceptibility to these disorders, understanding the neural regulation of aggression and social status will have significant import for translational studies.

## Aggression

Aggression is a complex phenomenon that has many different forms and adaptive functions (1–4). Aggression has been classified in a variety of ways, but the three forms of aggression that have been studied most extensively and that may be most closely linked with reproductive success are offensive aggression, defensive aggression, and parental aggression (the most frequently studied form of parental aggression is maternal aggression). Offensive aggression has been defined as aggression involving response to challenge over important resources; defensive aggression as attack in defense of an individual’s own integrity; and maternal aggression as aggression aimed at intruders threatening the offspring of a lactating female. As noted by Blanchard et al. (5), “the distinction between offensive and defensive aggression is based on a number of aspects of behavior, including antecedent conditions, organismic variables, attack topography (target of attack on the opponent’s body), and typical outcomes.” The ethograms for offensive and defensive aggression are unique (6). Maternal aggression appears to include elements of both offensive and defensive aggression and probably represents a mixed category (7). Therefore, based on these criteria, the current consensus is that offensive, defensive, and maternal aggression represent different categories of aggression, although similar behaviors can, at times, be observed in all three categories. In this review, we will focus on the role of arginine-vasopressin (AVP) in mammalian offensive aggression and how this role relates to the formation and maintenance of social relationships [for reviews of the role of AVP in maternal aggression see Ref. (7–9)]. When aggression occurs during a social encounter between strangers, both offensive and defensive aggression is observed. Winners and losers are rapidly determined and winners become dominant and losers subordinate. Along with the formation of dominant/subordinate relationships, aggressive behavior declines and other agonistic behaviors that serve to reinforce social status occur in increasing frequency (e.g., social communication). In the second section of this review, the role of AVP in the formation and maintenance of social status will be discussed.

### AVP and the Neural Control of Offensive Aggression in Males

**Figure 1:**
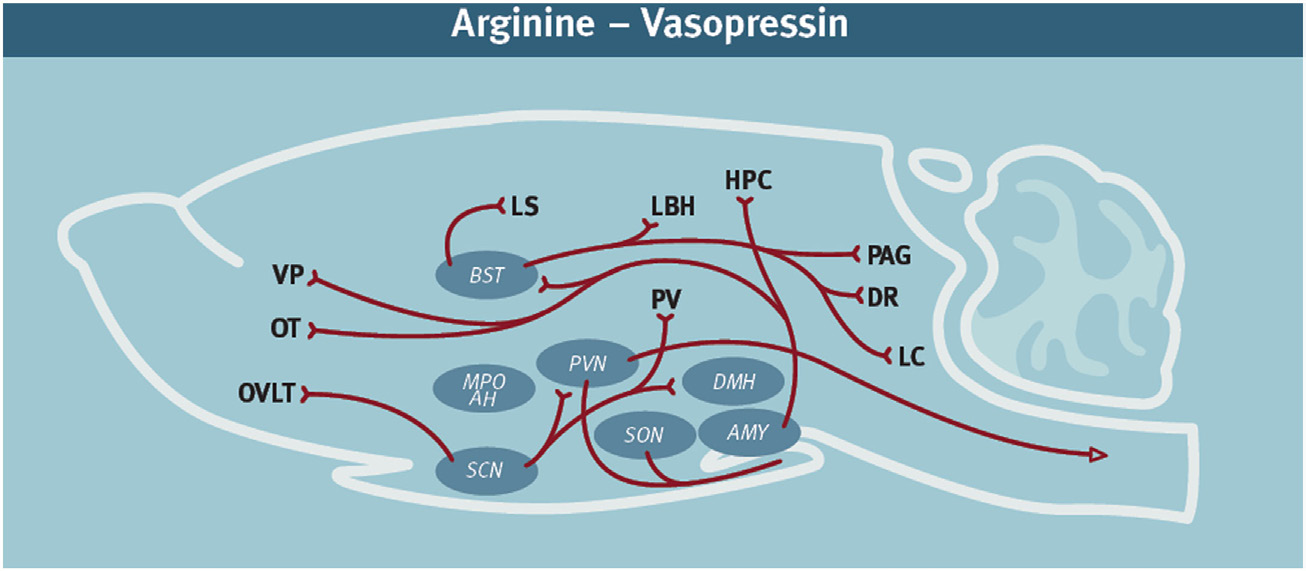
Diagram of the arginine-vasopressin (AVP)-containing neural network in rodents. It is noteworthy that AVP immunoreactivity can vary by species, sex, age, and social experience (16, 17). These diagrams represent a compilation of the major AVP projections from several rodent species. In addition to the cell bodies indicated in the diagram, there are also accessory nuclei that likely also play an important role. AVP network (11, 18–23). Abbreviations: AMY, amygdala; DR, dorsal raphe; HPC, hippocampus; LS, lateral septum; LC, locus coeruleus; MPO AH, medial preoptic area—anterior hypothalamus; OT, olfactory tubercle; OVLT, organum vasculosum laminae terminalis; PVN, paraventricular nucleus; PAG, periaqueductal gray; PV, periventricular nucleus hypothalamus; SCN, suprachiasmatic nucleus; SON, supraoptic nucleus; VP, [figure modified from Ref. (24) with permission].

Historically, AVP has been well known for its many physiological actions in the periphery, including water reabsorption and cardiovascular homeostasis (10). More recently, however, AVP has been identified as a critical neurochemical signal in the neural circuitry regulating offensive aggression. Neuroanatomical studies have demonstrated that in addition to the well-known AVP-containing hypothalamic projections to the neurohypophysis, there is an extensive neural network of AVP-containing projections throughout the brain (11–13) (Figure 1). For example, in Syrian hamsters, AVP neurons originating from hypothalamic nuclei, such as the medial supraoptic nucleus (mSON), nucleus circularis (NC), and paraventricular nucleus (PVN), project to many brain regions thought to be involved in regulating social behavior. There is increasing evidence that this AVP circuitry is engaged during social encounters. During agonistic encounters, enhanced activation of AVP-containing neurons in the mSON and NC is observed in male hamsters compared to controls (13). Cross-fostering male California mice with the less aggressive and less territorial white-footed mouse reduces adult aggression and the amount of AVP- immunoreactivity (ir) in the mSON and the bed nucleus of the stria terminalis (BNST) (14). After an agonistic encounter between female pigs the expression of AVP mRNA is enhanced in the medial amygdala, septum, and BNST of aggressive compared to non-aggressive individuals (15).

The first evidence that AVP is involved in the control of aggression came from the finding that injection of an antagonist of the V1a AVP receptor (V1aR) into the anterior hypothalamus (AH) of male hamsters inhibits aggression (25, 26). Subsequent studies in hamsters and voles have found that AVP administered within the AH can stimulate high levels of aggression (27–30). Interestingly, however, the ability of AVP to stimulate aggression within the AH appears to depend on an upregulation in the number of V1aRs in the AH. This upregulation appears to occur as the result of specific types of social experience. For example, AVP is effective in increasing aggression following its injection into the AH in male hamsters that had previously been trained to fight but not in hamsters housed in stable social groups (27, 31). Socially isolated male hamsters also display higher levels of aggression and greater numbers of V1aRs in the AH than socially housed males (32, 33).

The upregulation of V1aRs in the AH also appears to be important in AVP regulation of male aggression in voles. Following pair bonding, male prairie voles engage in high levels of aggression and have significantly more V1aRs in the AH than sexually naïve voles (29, 34). There is also significantly more aggression and higher levels of AVP released in the AH in pair bonded males exposed to novel females than in males exposed to their female partners. It has been suggested that AVP is necessary for the transition to increased aggressiveness following pair bonding but not for the expression of aggressive behavior (34). In support of this idea is the finding that V1aR antagonists block the induction of partner preference but not the expression of aggression displayed by breeder male voles with established selective aggression (34). On the other hand, induction of large numbers of V1aRs in the AH by viral vector-mediated gene transfer significantly increases aggression in non-pair bonded males (29). In summary, social experience can modulate the number of V1aRs in the AH and the number of V1aRs in the AH can regulate the amount of male offensive aggression. Nevertheless, the precise role of V1aRs in regulating aggression remains to be determined and it is important to note that high levels of aggression can occur in the absence of V1aR activation at least in males with prior aggressive experience.

Further support for the role of AVP and V1aRs in the control of aggression comes from studies examining how drugs of abuse can stimulate aggression. Anabolic steroids (AAS) administered to adolescent male hamsters produce high levels of aggression that is inhibited by a V1aR antagonist injected into the AH (35). AAS also increases AVP fiber density and content within the AH without altering V1aR binding (36–38). Conversely, after 18 days of withdrawal from AAS, there is a reduction in both aggressive behavior and AVP fiber density in AAS-treated male hamsters (39). Chronic, low-dose cocaine treatment during adolescence increases adulthood aggression and electrically stimulated AVP release in the AH of male hamsters (40, 41). In male prairie voles, administration of amphetamine increases aggression and increases V1aR binding in the AH (29). In summary, drugs of abuse can act within the AH to alter aggression by influencing the amount of AVP innervation and/or by altering the number of V1aRs.

Another hypothalamic region where AVP plays an important role in male aggression is the ventrolateral hypothalamus (VLH). Injection of AVP into the VLH increases aggression in gonadally intact male hamsters and castrated males given testosterone but not in castrated males without testosterone replacement (42). The effects of testosterone on AVP-stimulated aggression are likely mediated by the effects of testosterone on V1aR number in the VLH. Castration reduces V1aR binding in the VLH and pre-castration levels of V1aR binding can be restored by the administration of testosterone (43–45).

There is also evidence for a relationship between aggression and AVP in other regions of the limbic system. In male California mice, aggression is positively correlated with the number of AVP-ir neurons in the posteromedial BNST (46). In the septum, a negative correlation between AVP fiber density and male aggression has been observed in strains of rats and mice bred for varying levels of aggression (47, 48). In other studies, employing rats selected for low or high anxiety, the release of AVP in the septum is considerably lower in the much more aggressive low anxiety rats than in the less aggressive, high anxiety rats. Interestingly, however, injection of AVP or a V1aR antagonist into the septum does not alter the expression of aggression in the low aggressive or high aggressive rats (49). Thus, although relationships between aggression and AVP-ir and release exist in the LS, there is no direct evidence that AVP acts in the septum to modulate aggression.

Gene targeting approaches have also been used to study the role of AVP receptors in male aggression. Surprisingly complete knockout of V1aRs has no effect on aggression in male mice and produces only a slight deficit in olfaction (50). By contrast, knockout of AVP V1b receptors (V1bR) significantly reduces aggression (51, 52). Further the reduction in offensive aggression in V1b knockout mice is due to deficits in social motivation and not in deficits in olfactory function (53, 54). Interestingly, knockout of the V1bR gene produces deficits specific to forms of aggression directed toward a conspecific (55). Further support for a role of V1bRs in aggression come from pharmacological studies where peripheral administration of a V1bR antagonist reduced aggression in male hamsters (56). V1bRs in the hippocampus likely play a prominent role regulating aggression. When V1bR function is restored in the CA2 region of the hippocampus in male knockout mice by lentiviral delivery of the V1bR gene, offensive aggression is partially restored (57). The ability of mice to express aggression in the absence of V1aRs is surprising given the considerable pharmacological data in other species indicating the importance of V1aRs in male aggression. The simplest explanation for the continued aggressiveness of these knockout mice might be a developmental compensation for the life-long loss of V1aRs. Perhaps the V1bR acts as such a compensatory mechanism that preserves aggressive behavior in the V1aR knockout mice.

### AVP and the Neural Control of Offensive Aggression in Females

Little is known about the neurobiology of offensive aggression in females. The emphasis of competitive behaviors, such as offensive aggression in sexual selection in males, has resulted in little attention being paid to the neural control of offensive aggression in females (58). It is clear, however, that females as well as males compete for resources and mates to achieve reproductive benefits and that female competition has a significant role in evolution in mammals (59–61). Females compete for resources such as food, nest sites, and protection using a number of different strategies, including intergroup aggression, dominance relationships, and territoriality. As such, there are similarities in many of the competitive behaviors expressed by males and females (e.g., offensive aggression). Although many of the competitive behaviors displayed by males and females are similar, the neural mechanisms controlling them may be fundamentally different. Evolutionary biology would suggest that the social strategies used by females and males evolved in response to very different selective pressures. As a result, it seems likely that some of these behavioral similarities arose as the result of convergent evolution in the neural mechanisms controlling social behavior. Indeed, the likelihood that there are sex differences in the neural mechanisms regulating aggression highlight the importance of a better understanding of how these mechanisms function in males and females. Given the substantial sex differences in the incidence of many psychiatric disorders, understanding the sex differences in the neural mechanisms regulating social behavior has the potential for substantial translational significance (62).

Another reason for the absence of data on the neurobiology of offensive aggression in females is the choice of species used to study the physiological mechanisms controlling aggression. Studies of the physiology of aggression have been conducted primarily in laboratory rats and mice (5, 7). Because laboratory rats and mice rarely display female offensive aggression, few studies on the physiological regulation of these critically important forms of aggression have been conducted (63, 64). Another laboratory species, Syrian hamsters, provide an outstanding model with which to study female competitive behavior. Female hamsters, like many female primates, display a number of different competitive strategies such as the expression of spontaneous offensive aggression, the rapid formation of hierarchical dominance relationships, and the ability to inhibit the reproductive capacity of other females (65–67).

Studies in hamsters provide the first evidence that there are fundamental sex differences in the neural circuitry controlling offensive aggression and that some of these sex differences involve AVP. AVP in the AH has opposite effects on the expression of aggression in males and females. Injection of AVP into the AH of female hamsters reduces aggression, whereas injection of a V1aR antagonist in female hamsters increases aggression (68, 69). While social isolation increases aggression in female hamsters, as it does in male hamsters, there is no increase in V1aR density in female hamsters as there is in males (33).

There are also some interesting sex differences in the developmental effects of AVP on adult aggression. AVP administered during the early postnatal period influences the expression of adult aggression in a sex-dependent manner (70, 71). Male but not female prairie voles exhibit significantly higher levels of aggression as adults when administered AVP peripherally during the early postnatal period. Taken together, the existing data indicate that AVP can play a critical, but opposite role in the regulation of aggression in males and females.

There is only a limited amount of data on the role of AVP in aggression in primates. In chimpanzees, polymorphisms of the V1aR gene are associated with enhanced or reduced aggressive behaviors (72). In humans, AVP cerebrospinal fluid levels positively correlate with a life history of aggression in individuals who meet DSM-IV criteria for personality disorder (73). Other studies in humans while not directly addressing the effects of AVP on aggression have identified some striking differences in the effect of AVP on social cognition. Intranasal administration of AVP in humans has sex-dependent effects on the social valence of stimuli (74). In women, AVP enhanced the perception of friendliness in the faces of unfamiliar women and stimulated affiliative facial motor patterns. In men, AVP reduced the perception of friendliness in the faces of unfamiliar men and stimulated agonistic facial motor patterns.

### AVP Interactions with Other Neurochemical Signals in the Control of Aggression

Arginine-vasopressin interacts with several neurochemical signals to regulate offensive aggression. In males, serotonin (5-HT) has potent inhibitory effects on the expression of aggression in species ranging from fish to primates and at least some of these effects of 5-HT are mediated by its interactions with AVP (30, 75). One site where AVP and 5-HT likely interact to regulate aggression is the AH. The AH receives AVP-containing projections from the mSON and NC as well as 5-HT-containing projections from the raphe (13, 76). Co-infusion of AVP and the 5-HT1a receptor (5-HT1aR) agonist 8-OH-DPAT into the AH of male hamsters produces higher levels of aggression than 8-OH-DPAT alone and lower levels of aggression than AVP (69, 77). Systemic administration of the selective serotonin reuptake inhibitor, fluoxetine, decreases aggression in male hamsters (27) and pre-treatment with fluoxetine blocks AVP-induced aggression in both the AH and VLH (42, 77, 78). Chronic administration of fluoxetine during male adolescence increases both adulthood aggression and AVP fiber innervation in the AH (79, 80).

Surprisingly, although the effects of 5-HT have been investigated in hundreds of studies in males, not until recently have the effects of 5-HT been examined in females (81, 82). By contrast, to the striking inhibitory effects of 5-HT on aggression in males, injection of 8-OH-DPAT into the AH produces a dose-dependent increase in aggression in females. Co-infusion of AVP and 8-OH-DPAT into the AH of female hamsters produces lower levels of aggression than 8-OH-DPAT alone and higher levels of aggression than AVP (69, 77). Taken together, these studies indicate that AVP and 5-HT act in opposite ways within the AH to regulate offensive aggression in males and females.

Oxytocin (OT) is very similar in structure to AVP sharing seven of nine amino acid sequences (83, 84). In addition, OT and AVP receptors are very similar in structure and can respond in a relatively unselective manner to both neuropeptides (24). Indeed, OT can have effects on aggression similar to those of AVP. Like AVP, OT injected into the AH reduces aggression in a dose-dependent manner in female hamsters (85). Injection of an OT antagonist into the AH increases aggression in female hamsters (85). Total OT knockout male and female mice have higher levels of aggression than controls as do total male OT receptor (OTR) knockouts (86–88). There is increasing evidence that OT and AVP can influence a number of different social behaviors by acting on each other’s receptors. OT or AVP induce social communication behavior by activating V1aRs but not OTRs, while OT or AVP can enhance social recognition and social reward by activating OTRs but not V1aRs (24, 89–92). While both OT and AVP can influence the expression of aggression the roles of OT and AVP receptors in mediating these effects remains to be fully understood.

Glutamate is also capable of interacting with AVP in the neural control of aggression. In a model of heightened male aggression in which male hamsters are chronically exposed to AAS during adolescence, glutamate receptor subunit type 1 density is increased in the VLH and vesicular glutamate transporter 2 density is increased in the AH, two brain regions where AVP acts to control aggression (93, 94). Interestingly, AVP can induce the release of glutamate from astrocytes through binding of both V1aRs and V1bRs (95). AVP-induced release of glutamate provides a potential mechanism through which offensive aggression could be facilitated within the AH and VLH.

## Social Status

For most mammalian and non-mammalian species, the formation and maintenance of dominance relationships rely on agonistic behaviors, particularly aggression and social communication. Although aggression has been commonly characterized as a negative social interaction, aggression plays a very constructive role in the formation of these important social relationships. In nearly all mammals, dominance relationships represent social relationships that have many adaptive functions (e.g., resource distribution) ultimately resulting in a reduction of social conflict (96). Most dominance relationships are determined rapidly by aggression but are primarily maintained by social communication (e.g., scent marking, vocalization, non-contact aggression or harassment, etc.) thereby reducing the dangers of continual, intense conflict (97). Success in maintaining these relationships depends on social skills that are also hallmarks of psychiatric health, such as effective social communication. If, for example, social communication is dysfunctional, then the social interactions become maladaptive, resulting in continuously high levels of social conflict (98). Importantly, dominance relationships have different consequences for the winners and the losers. Winning is rewarding (99, 100) and losing is stressful (101), particularly if subordinate status is imposed continually over time (102, 103). Indeed, losing is known to be a potent and ethologically relevant stressor and has become a leading model for investigation of the neural circuits and behavioral phenotypes (104) that are activated by social stress (105–107) and that have been widely shown to be useful models of stress-related mood and anxiety disorders (108–110).

### AVP and the Neural Control of Social Status in Males

**Figure 2:**
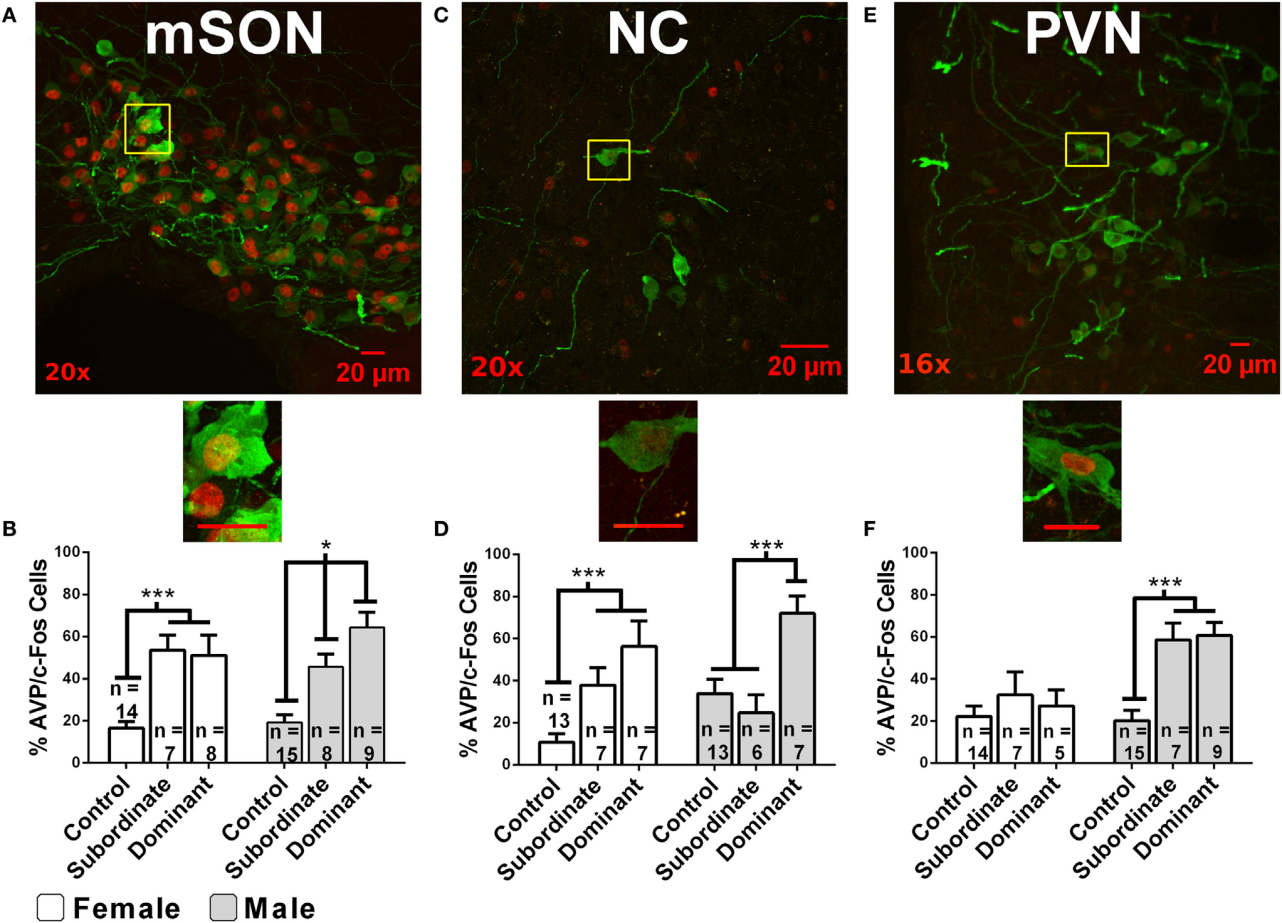
Immunofluorescent colocalization of AVP-ir (green) and fos-ir (red) in cells within the following hypothalamic nuclei: the medial supraoptic nucleus [mSON; **(A,B)**], nucleus circularis [NC; **(C,D)**], and medial paraventricular nucleus [PVN; **(E,F)**]. Magnification is indicated in the bottom left corner. Yellow boxes indicate subregions magnified to 40×. Scale bars are 20 µM. Graphs indicate the percentage of AVP-ir cells that colocalize with fos-ir cells (% of activated AVP cells) as a function of acquired dominance status and sex in the mSON, NC, or PVN. White arrows indicate AVP-ir cells colocalized with fos-ir. Error bars indicate SEM. * indicates *p* < 0.05 and *** indicates *p* < 0.01 [figure modified from Ref. (69) with permission].

Social relationships among animals commonly take the form of hierarchical dominance relationships. Although the focus of this review is on the role of AVP in social status in mammals, there is considerable evidence that the non-mammalian homolog of AVP, arginine-vasotocin plays an important role in dominance relationships in birds, reptiles, amphibians, and fish [for reviews, see Ref. (111–115)]. Given its role in aggression, it is not surprising that AVP is also involved in the acquisition and maintenance of dominance relationships. In male hamsters, AVP-ir neurons in the mSON and NC display significantly higher levels of activation (as indicated by increased AVP-ir/fos-ir) in winners (i.e., dominants) than in losers (i.e., subordinates) or controls after a single agonistic encounter (69) (Figure 2). Differences in social status are associated with differences in AVP-ir and V1aR binding in the hypothalamus. Subordinate male hamsters in stable dominance relationships have significantly fewer AVP-ir neurons in the NC compared to dominant and control males (116) and dominant male hamsters have significantly more V1aR binding than subordinate or control hamsters in the hypothalamus (117). V1bR knockout male mice can establish dominance hierarchies suggesting that V1bRs may not be essential for the formation of these relationships. Non-dominant V1bR knockout mice do, however, engage in less offensive aggression than wild-type controls (118).

Social status is associated with different amounts of AVP-ir in several hypothalamic regions. In mandarin voles, dominant males have significantly more AVP-ir in the PVN, SON, LH, and AH than subordinates (119). Interestingly, no differences were observed in OT-ir between dominant and subordinate male voles in any of these brain regions except the PVN where OT-ir was lower in dominant than in subordinate males. By contrast, in male mice subordination seems to be associated with higher levels of AVP-ir in the PVN. In male California mice, a single social defeat significantly increases the activation of AVP neurons (i.e., AVP-ir/fos-ir) in both the PVN and SON (46). In addition, more than 10 weeks after social defeat AVP mRNA and AVP-ir were significantly reduced in the PVN. In an inbred strain of mice, chronic defeat increases AVP mRNA expression in the PVN of males (120). Adolescent male hamsters that are chronically defeated are more aggressive in adulthood toward smaller, weaker hamsters and less aggressive toward larger, stronger hamsters (121). Social subjugation also results in a 50% reduction in AVP-ir fiber density in the region of the AH involved in regulating aggression (121).

Several studies have also examined the effects of the porcine form of vasopressin, lysine-vasopressin (LVP), on dominant and subordinate behavior. LVP differs from AVP in that lysine is substituted for arginine in the eighth amino acid position. In male mice, administration of LVP increases submissive behavior when given prior to social defeat, immediately after social defeat, or immediately prior to a social interaction the day following social defeat (122, 123). By contrast, LVP administration prior to social defeat has no effect on dominance behavior (122–124). Interestingly, LVP binds to OTRs with a higher affinity than to AVP receptors suggesting the possibility that the ability of LVP to enhance submissive and not dominance behavior is mediated by OTRs (125).

**Figure 3:**
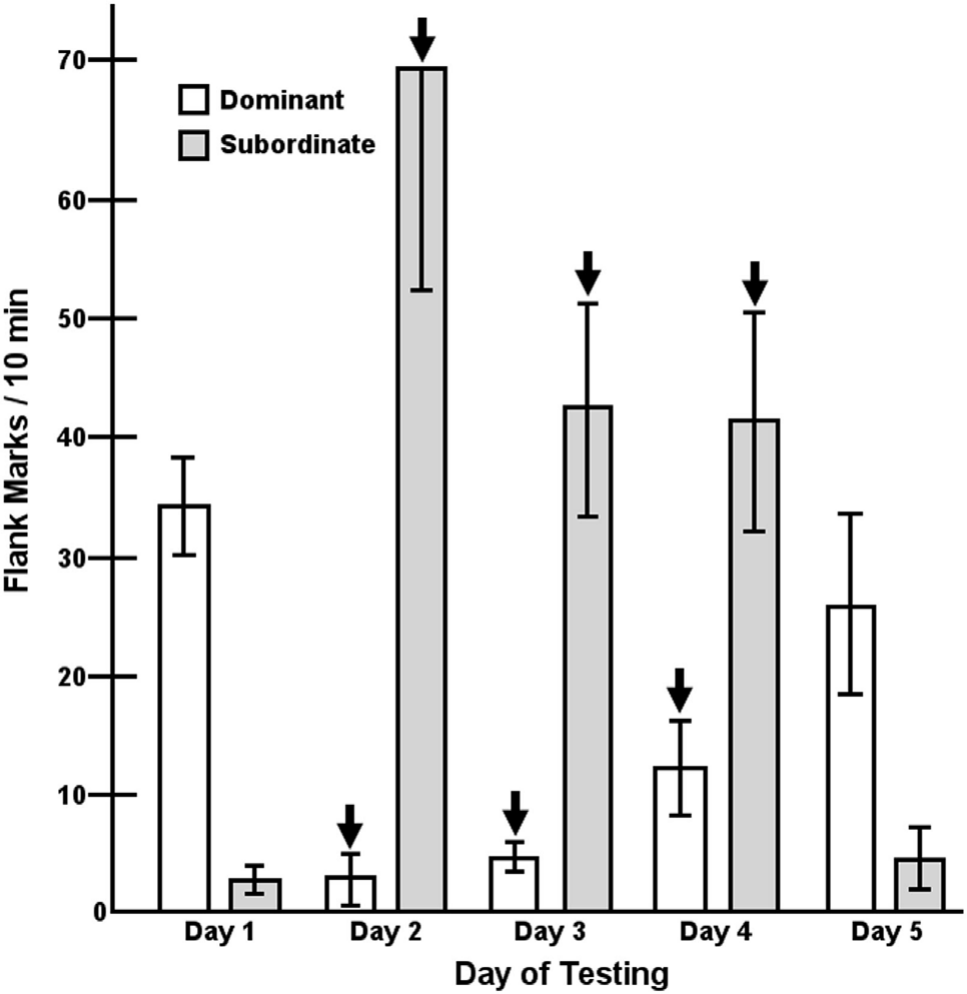
Flank marking in pairs (*n* = 5) of dominant/subordinate male hamsters in response to the microinjection of dPTyr(Me)AVP [V1a receptor (V1aR) antagonist] and arginine-vasopressin into dominant and subordinate members, respectively, over three consecutive days (arrows, days 2–4). Each bar represents the mean ± SEM of the number of flank marks made in a 10-min test over five consecutive days. A two-way analysis of variance resulted in a significant treatments by trials interaction, *F*(4,32) = 24.04, *p* < 0.001 [figure modified from Ref. (98) with permission].

A key element in the maintenance of dominance relationships is effective social communication (3, 97, 98). In hamsters, an important form of social communication is a type of scent marking called flank marking (126). Flank marking is used to communicate a variety of socially important information including social status (98, 126, 127). AVP plays a critical role in regulating flank marking by its actions within the hypothalamus as well as several other structures including the lateral septum and periaqueductal gray (128–132). Indeed, injection of AVP into the hypothalamus induces high levels of flank marking and injection of a selective V1aR antagonist blocks the expression flank marking (90, 133–136). After an initial agonistic encounter between two hamsters where dominance is defined by the winner of aggressive interactions, the levels of aggression rapidly decline and the expression of flank marking increases (98). Interestingly, flank marking increases in both the dominant and subordinate hamster, although the amount of flank marking is substantially higher in the dominant. When the dominant hamster’s flank marking is inhibited by hypothalamic injection of a V1aR antagonist the subordinate increases its flank marking (137). Interestingly, inhibition of flank marking in dominant hamsters by the injection of a V1aR antagonist while simultaneously stimulating flank marking in the subordinate by the injection of AVP for several consecutive days does not produce a reversal of social status. On the day immediately after the injections are terminated, the initially dominant hamster again flank marks significantly more than its subordinate partner (Figure 3). Thus, while flank marking communicates dominance status, it does not determine dominance status.

### AVP and the Neural Control of Social Status in Females

Comparatively little is known about the role of AVP in the neural mechanisms regulating dominance relationships in females. One approach has been to examine the relationship between the numbers of AVP-ir neurons and social status. In mandarin voles, dominant females have significantly more AVP-ir cells in the AH and LH than subordinates (119). No differences were found in the number of AVP-ir cells in either the PVN or SON. Significant sex differences in the number of AVP-ir cells were also observed. Dominant females have significantly fewer AVP-ir cells in the PVN, SON, AH, and LH than dominant males and subordinate females have significantly lower levels of AVP-ir than subordinate males. No differences were observed in OT-ir between dominant and subordinate females in any of the brain regions examined.

Another approach has been to examine the relationship between social status and the activation of AVP-containing neurons. Following social defeat, female California mice, like males, have significantly more activated AVP neurons (i.e., AVP-ir/fos-ir) in both the SON and PVN (46). While the acute of effects of defeat are similar in females and males the long term effects of defeat on AVP neurons is stronger in males than in females. In hamsters, neuronal activation in AVP-containing neurons (i.e., AVP-ir/fos-ir) in the NC and mSON is higher in both male and female hamsters following an agonistic encounter than in controls (Figure 3). In females, however, similar levels of activation are seen in dominant and subordinate hamsters, while dominant male hamsters display significantly higher levels of activation as compared to subordinate males (69). Thus, there is a substantial sex difference in the relationship between social status and the activation of AVP-containing neurons within the hypothalamus.

Arginine-vasopressin plays an important role in the communication of social status in female hamsters as it does in males (3). Perhaps surprisingly given the opposite effects of AVP on aggression in the AH in males and females, AVP induces flank marking at high levels in both males and females (133). Indeed, the dose–response relationship between the hypothalamic injection of AVP and flank marking are almost identical in male and female hamsters (138). It, therefore, seems likely that although V1aRs mediate the effects of AVP on aggression and flank marking there are important differences in the circuitry controlling these behaviors. Whether separate populations of V1aRs in the AH control aggression and flanking marking is not known, there is some evidence to support this possibility. Exposure to short “winter-like” photoperiods significantly reduces, but does not eliminate V1aR binding in the AH of male hamsters (139). Interestingly, short photoperiod significantly reduces the ability of AVP injected into the AH to increase aggression but has no effect on the ability of AVP to induce flank marking (28, 139). As such, there may be a short photoperiod sensitive subpopulation of V1aRs in the AH that mediate aggression and a short photoperiod in-sensitive subpopulation of V1aRs in the AH that mediate flank marking.

### AVP Interactions with Other Neurochemical Signals in the Control of Dominance

Several neurochemical signals can interact with AVP to modulate the communication of social status. In female hamsters, injection of norepinephrine significantly reduces AVP-induced flank marking by its actions in the AH (140). In male hamsters, injection of galanin into the AH significantly reduces AVP-induced flank marking (141, 142). Glutamate receptors within the AH appear to be necessary for the induction of flank marking by AVP at least in male hamsters (143). Co-administration of the glutamate receptor antagonists AP-5 or GAMS significantly inhibits the expression of AVP-induced flank marking. Further support for the hypothesis that glutamate has a critical role in AVP-induced flank marking comes from evidence that AVP can induce glutamate release from astrocytes (95).

There is considerable evidence that 5-HT can modulate AVP-induced flank marking. As discussed earlier, both AVP- and 5-HT-containing projections terminate in the AH so the AH is a likely site for AVP–5-HT interactions (13, 76). When 5-HT or 5-HT agonists are combined with AVP and injected into the AH, the ability of AVP to stimulate flank marking is substantially reduced in both males and females (142, 144, 145). The 5-HT receptor subtypes mediating the effects of 5-HT on flank marking in the AH are not fully defined but there is evidence for the potential involvement of 5-HT1a, 5-HT1b, 5-HT7, and 5-HT4 receptors (146). Systemic administration of fluoxetine can also reduce flank marking produced by injection of AVP into the AH or VLH so 5-HT could be acting a multiple brain sites to inhibit flank marking (144, 147).

The raphe likely plays an important role in mediating how AVP and 5-HT interact to regulate social status given its substantial projections to the AH. Indeed, in female hamsters, 5-HT-ir neurons in the dorsal raphe (DRN) display significantly higher levels of activation (as indicated by increased 5-HT-ir/fos-ir) in winners (i.e., dominant) than in losers (i.e., subordinates) or controls after a single agonistic encounter (69). By contrast, a similar relationship between social status and the activation of 5-HT neurons in the DRN is not seen in males. Despite these major sex differences, there is also a more nuanced relationship between the activation of DRN neurons and social status in males and females. Subordinate males, but not females, display more activation of 5-HT neurons in the ventral subregion of the anterior DRN than dominant or control males and dominance status alters the activation of 5-HT neurons in the dorsal subregion of the anterior DRN in both males and females (148).

There is additional support for a relationship between 5-HT and social status in males. 5-HT1aR mRNA is higher in the DRN of dominant male hamsters in established dominance relationships compared to subordinates (148). Injection of a 5-HT1aR agonist into the DRN of male hamsters, either prior to social defeat or prior to testing for conditioned defeat, reduces submissive and defensive behaviors and infusion of a 5-HT1aR antagonist into the DRN increases submissive and defensive behaviors (149). Systemic injection of a 5-HT2a receptor agonist in male hamsters after social defeat and prior to testing for conditioned defeat decreases submissive behavior toward a non-aggressive stimulus animal (150). Taken together, the DRN is involved in regulating social status in both males and females, although there are major sex differences in the activation of 5-HT neurons during the formation of dominance relationships.

## Summary and Conclusions

Arginine-vasopressin plays an important role in the establishment of dominance relationships *via* its effects on aggression and in the maintenance of dominance relationships by its effects on social communication. Although much remains to be learned about the neural circuitry controlling aggression and social status, the hypothalamus is a key site for the actions of AVP. V1aRs in the hypothalamus appear to be critical for the modulation of aggression and for social communication by AVP. The number of V1aRs in the hypothalamus can be influenced by a variety of factors including social experience and gonadal hormones. In males, AVP-induced aggression appears to require an upregulation of V1aRs. Although the most direct evidence for a role of AVP in modulating aggression has been in the hypothalamus there is a relationship between AVP and aggression in several other limbic structures, including the BNST and septum. Although AVP has a substantial role in regulating male aggression it is not clear if AVP promotes aggression in the same manner in all circumstances. For example, it remains to be determined if AVP drives male aggression directly and/or whether AVP is necessary for the transition from a non-aggressive state to aggressiveness.

In females, there have been fewer studies examining the role of AVP in regulating aggression. Nevertheless, there appear to be striking sex differences in the hypothalamic effects of AVP on aggression. While AVP stimulates aggression in males, it inhibits aggression in females. Despite the opposite effects of AVP on aggression in males and females the effects of AVP on aggression are mediated by V1aRs in both sexes. There are also sex differences in how V1aRs can be regulated in the hypothalamus. In males, social isolation increases the number of V1aRs in the AH, but in females social isolation does not change the number of V1aRs despite the fact that social isolation increases aggressiveness in both males and females. Interestingly, the hypothalamic effects of 5-HT on aggression complement the effects of AVP. While activation of 5-HT1aRs in the AH inhibits aggression in males, activation of these receptors stimulates aggression in females.

While aggression plays a critical role in the formation of dominance relationships, it becomes less important after the winners and losers have been determined. In most cases, dominance relationships remain comparatively stable over time as communication is used as a “reminder” of an individual’s social status. In hamsters AVP has a central role in the social communication of dominance status. Interestingly, although AVP has the opposite effects on aggression in males and females, AVP potently stimulates flank marking in both males and females. These similarities and differences in the effects of AVP on flank marking and aggression are particularly interesting because both appear to be mediated by V1aRs. It seems likely that different subpopulations of V1aRs mediate the effects of AVP on aggression and flank marking even though they are found in overlapping regions of the hypothalamus. It is also interesting that 5-HT has the same inhibitory effects on AVP-stimulated flank marking in males and females.

It has been proposed that aggressiveness, dominance, and active coping strategies may represent traits that result in more resistance to psychiatric disorders (151, 152). In addition, a role for the vasopressinergic system in psychiatric disorders is receiving increasing attention (153). Importantly, however, sex differences in the effects of AVP on social behavior and in particular on aggression have received little attention. Given the substantial sex differences in the effects of AVP and 5-HT on aggression and dominance and the dramatic sex differences seen in the incidence of psychiatric disorders, it will be important to determine the extent to which these phenomena are linked. Further examination of the AVP system as a target for clinical intervention is particularly timely because of the advent of AVP-active drugs that can be administered orally (154, 155).

## Author Contributions

JIT, CFF, and HEA all wrote and revised this manuscript.

## Conflict of Interest Statement

The authors declare that the research was conducted in the absence of any commercial or financial relationships that could be construed as a potential conflict of interest.
